# Assessment of multiresolution segmentation for delimiting drumlins in digital elevation models

**DOI:** 10.1016/j.geomorph.2014.02.028

**Published:** 2014-06-01

**Authors:** Clemens Eisank, Mike Smith, John Hillier

**Affiliations:** a Department of Geoinformatics-Z_GIS, University of Salzburg, Schillerstraße 30, 5020 Salzburg, Austria; b School of Geography, Geology and Environment, Kingston University, KT1 2EE, UK; c Department of Geography, Loughborough University, LE11 3TU, UK

**Keywords:** Land-surface segmentation, Object-based image analysis (OBIA), Synthetic drumlins, Geomorphometry, Supervised, Region-growing

## Abstract

Mapping or “delimiting” landforms is one of geomorphology's primary tools. Computer-based techniques such as land-surface segmentation allow the emulation of the process of manual landform delineation. Land-surface segmentation exhaustively subdivides a digital elevation model (DEM) into morphometrically-homogeneous irregularly-shaped regions, called terrain segments. Terrain segments can be created from various land-surface parameters (LSP) at multiple scales, and may therefore potentially correspond to the spatial extents of landforms such as drumlins. However, this depends on the segmentation algorithm, the parameterization, and the LSPs. In the present study we assess the widely used multiresolution segmentation (MRS) algorithm for its potential in providing terrain segments which delimit drumlins. Supervised testing was based on five 5-m DEMs that represented a set of 173 synthetic drumlins at random but representative positions in the same landscape. Five LSPs were tested, and four variants were computed for each LSP to assess the impact of median filtering of DEMs, and logarithmic transformation of LSPs. The testing scheme (1) employs MRS to partition each LSP exhaustively into 200 coarser scales of terrain segments by increasing the scale parameter (*SP*), (2) identifies the spatially best matching terrain segment for each reference drumlin, and (3) computes four segmentation accuracy metrics for quantifying the overall spatial match between drumlin segments and reference drumlins. Results of 100 tests showed that MRS tends to perform best on LSPs that are regionally derived from filtered DEMs, and then log-transformed. MRS delineated 97% of the detected drumlins at *SP* values between 1 and 50. Drumlin delimitation rates with values up to 50% are in line with the success of manual interpretations. Synthetic DEMs are well-suited for assessing landform quantification methods such as MRS, since subjectivity in the reference data is avoided which increases the reliability, validity and applicability of results.

## Highlights

• Multiresolution segmentation is tested for delimiting drumlins in synthetic DEMs.
• Drumlins may be delimited by individual terrain segments.
• Recommendations for optimizing terrain-based drumlin delimitation are given.
• Ideal terrain derivatives are regional, derived from a filtered DEM, and normalized.
• Recovery rates are similar to those achieved by experts in manual interpretation.

## Graphical abstract

**Figure:**
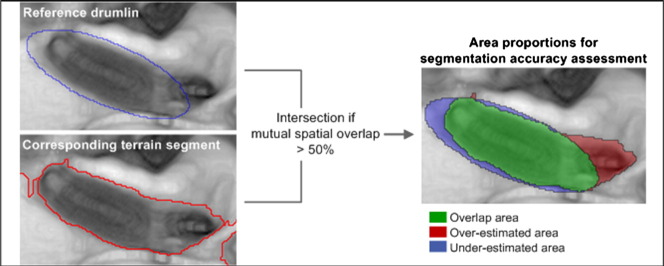

## 1 Introduction

In geomorphometry, land-surface segmentation is the process of exhaustively partitioning digital elevation models (DEMs) and derived land-surface parameters (LSPs; e.g. slope, curvature) into spatially discrete terrain segments (Minár and Evans, 2008). The available algorithms for land-surface segmentation can be split into two main groups (Romstad and Etzelmüller, 2012): edge-based and region-based. Edge-based algorithms identify topographic discontinuities such as slope breaks (e.g. Giles and Franklin, 1998; Matsuura and Aniya, 2012), or hydrological networks (e.g. Band et al., 2000; MacMillan et al., 2004) to structure the terrain surface into segments. Region-based methods construct terrain segments by merging adjacent grid cells of similar morphometric characteristics (e.g. Miliaresis, 2001). Detailed reviews on the topic of land-surface segmentation have been provided in recent studies (Minár and Evans, 2008; MacMillan and Shary, 2009; Drăguţ and Eisank, 2011; Romstad and Etzelmüller, 2012).

One of the widely used region-based algorithms for land-surface segmentation is multiresolution segmentation (MRS; Drăguţ and Blaschke, 2006; Van Asselen and Seijmonsbergen, 2006; Gerçek et al., 2011; Drăguţ and Eisank, 2012), as implemented in object-based image analysis (OBIA) software. MRS has been employed to delineate homogeneous terrain segments rather than landforms per se (e.g. Drăguţ and Blaschke, 2006; Drăguţ et al., 2011; Gerçek et al., 2011). Since the resultant segments are often significantly smaller than most targeted landforms, knowledge-based rules are specified to aggregate segments into larger spatial regions that correspond to the landform. Landforms that have been processed in such a way comprise alpine landforms (Van Asselen and Seijmonsbergen, 2006; Schneevoigt et al., 2008; Eisank et al., 2010), physiographic regions (Drăguţ and Eisank, 2012), and landslides (Van Den Eeckhaut et al., 2012). Although initial work towards automating the selection of knowledge-based aggregation rules has been conducted (Anders et al., 2011), trial-and-error approaches are still predominant (Gao et al., 2011; Laliberte et al., 2012).

Delimiting landforms as individual terrain segments by means of land-surface segmentation is attractive, since the time-consuming aggregation of terrain segments could be avoided, but remains challenging and a research frontier (Evans, 2012). MRS is potentially capable of automatically determining the edges of landforms (i.e. “delimit” them) in a procedure analogous to manually digitizing their outlines on-screen (e.g. Smith et al., 2006).

The landforms targeted in the present study are drumlins, elongated subglacial bedforms that classically resemble a half-buried egg with its long-axis horizontal. Due to postglacial erosion and anthropogenic modification, many drumlins deviate from this characteristic form (Clark et al., 2009). Since drumlins are generally bounded by concave breaks of slope, they should be extractable from a DEM using automated delimitation methods such as MRS (Evans, 2012). Drumlins have predominantly been mapped in the field (e.g. Smith et al., 2006) and/or manually delineated based on satellite images, aerial photographs and DEM visualizations (Livingstone et al., 2008; Clark et al., 2009; Smith et al., 2009). To date, few studies have attempted to automatically extract drumlins from DEMs. Saha et al. (2011) implemented a supervised object-based drumlin mapping approach based on medium-scale multi-band thematic layers and manually digitized references. d'Oleire-Oltmanns et al. (2013) employed a knowledge-based workflow to extract drumlins from relative relief. Both above-mentioned studies employed the MRS algorithm within an OBIA framework. Rutzinger et al. (2012) used a curvature map and user-specified thresholds to identify drumlin edges in a high-resolution DEM. Drumlins have also been semi-automatically extracted from contour maps by identifying closed-looped contours (Maclachlan and Eyles, 2013; McClenagan, 2013).

Instead of observed drumlins the present study investigates synthetic drumlins in a “real” DEM. These synthetic DEMs represent idealized drumlins in terms of 3D shape, and are representative datasets of an observed landscape. Hillier and Smith (2012) demonstrated that synthetic drumlins are well suited for evaluating landform quantification methods such as MRS, since the results can be interpreted objectively through the a priori knowledge of the drumlins.

The main objective of this study is to test the performance of the MRS algorithm with respect to the delimitation of synthetic drumlins based on DEMs and five LSPs. A supervised method is proposed that finds for each synthetic drumlin the spatially best matching terrain segment. Accuracy metrics quantify the overall spatial agreement between the synthetic drumlins and their corresponding terrain segments. In contrast to previous drumlin studies using OBIA (Saha et al., 2011; d'Oleire-Oltmanns et al., 2013), the focus is upon the MRS algorithm; the definition of classification rules is beyond the scope of this paper.

## 2 Background

This section provides details on the MRS algorithm, landform delimitation, and methods for optimizing MRS for landform delimitation.

MRS is a region-growing algorithm that performs iterative merging of adjacent segments (single grid cells in the first iteration) into larger segments. The merging stops when the growing segments exceed a user-specified threshold for the maximum homogeneity of LSP values for grid cells that form the segment (Baatz and Schäpe, 2000; Benz et al., 2004). The MRS algorithm can be applied to any gridded datasets including remote sensing, medical, and air-borne images (Blaschke, 2010). Drăguţ and Blaschke (2006) and Van Asselen and Seijmonsbergen (2006) introduced MRS to land-surface segmentation.

The intention is that terrain segments derived by MRS directly relate to the products of physical processes (e.g. Minár and Evans, 2008) such as landslides (Martha et al., 2011) or subglacial bedforms (d'Oleire-Oltmanns et al., 2013). Land-surface segmentation by means of MRS does not necessarily delineate landforms, thus “landform delimitation” can be seen as a special case of land-surface segmentation. Specifically, landform delimitation is the process of optimizing land-surface segmentation in such a way that individual terrain segments approximate the size and shape of landforms such as drumlins. Ideally, individual terrain segments perfectly match the spatial extent of landforms.

Two statistical approaches have been employed to optimize MRS towards landform delimitation. Drăguţ et al. (2010) devised an unsupervised method that applies a local variance for analyzing the inherent structure of gridded datasets based on iterative MRS to detect characteristic segmentation scales. Application of this method to DEMs and LSPs showed that terrain segments at detected scales may correspond to the size and shape of targeted landforms, i.e. physiographic regions in Drăguţ and Eisank (2012), and drumlins in d'Oleire-Oltmanns et al. (2013). Anders et al. (2011) proposed a supervised approach to optimize MRS for landform delimitation. In their multi-scale approach they compared frequency distributions of LSP cell values between reference polygons and terrain segments. Segmentation scales where the deviations between the distributions were minimal were selected as optimal. However, since the geomorphological reference was manually derived, some degree of uncertainty had to be accepted in this comparison.

## 3 Data and methods

### 3.1 Study area and synthetic DEMs

**Fig. 1:**
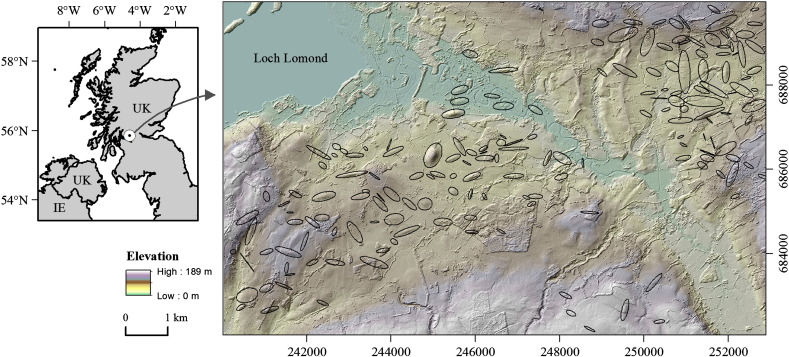
Study area located in Scotland, UK. The large map illustrates the relief-shaded synthetic 5-m DEM 1 with superimposed outlines of the randomly placed synthetic drumlins (*n* = 173). The DEM is colored according to elevation. Map coordinates are British National Grid.

The study area (Fig. 1) is a formerly glaciated region located in western central Scotland, in the north of the UK. It is approximately 13 km by 8 km in size.

Glacial landforms in the area have been mapped in the field (Rose and Smith, 2008), and two times, independently, based on DEMs (Smith et al., 2006; Clark et al., 2009). Landforms are of two ages: Younder Dryas [~ 12 ka] in the west and Last Glacial Maximum [~ 14.5 ka] in the east (Rose and Smith, 2008). At both times, ice flow has broadly been directed to the south and east (Sissons and Sissons, 1967; Rose, 1987).

The DEM used, for mapping, and in the creation of the synthetic DEMs was the NEXTmap Britain™ DEM or “NEXTmap”, as derived from single-pass interferometric synthetic aperture radar (IfSAR). This DEM is available as a grid at a horizontal spacing of 5 m, with vertical errors estimated to be less than 1 m (Intermap, 2004).

The conceptual and technical background for creating the synthetic DEMs for the study area has been described in detail in the original studies (Hillier and Smith, 2008; Smith et al., 2009; Hillier and Smith, 2012). However, for the sake of clarity, a summary of the steps involved is as follows:

1. Drumlin outlines were manually digitized from the original DEM. Then 60 m and 500 m median filters were applied to the DEM to isolate individual drumlins (Hillier and Smith, 2012), leaving their overall 3D shapes. These drumlins were removed from the original DEM to create a “drumlin-free” DEM.
2. Since the shape (height, width, length) of the extracted drumlins was approximately Gaussian, the synthetic drumlins were generated by idealizing the extracted drumlin 3D shapes based upon a Gaussian model (Hillier and Smith, 2012).
3. Synthetic DEMs were produced by inserting the idealized drumlin 3D shapes into the “drumlin-free” DEM. The locations of idealized drumlins were randomly determined (i.e. not at the locations of the originals), whilst respecting the overall spatial distribution and orientations of the original drumlins and topographic controls. By simulating different spatial distributions of the idealized drumlins, multiple synthetic DEMs for the study area were computed (Hillier and Smith, 2012).

For the present study five synthetic DEMs were tested. Specifically, these were DEMs 21 to 25 created using Mehtod 2 of Hillier and Smith (2012), here renamed DEM 1 to DEM 5. Each DEM represents the same set of 173 synthetic drumlins, distributed differently across the study area.

### 3.2 Preparation of DEMs and LSPs

The selection of land-surface parameters (LSPs) for landform delimitation using MRS is crucial. Numerous LSPs exist, and they can basically be divided into “local” and “regional” parameters (Olaya, 2009). Local LSPs are computed by applying either mathematical functions or statistical descriptors to DEM values inside a fixed-sized kernel (or window). Regional LSPs, and particularly those that relate to the hydrologic network, are valuable for landform extraction tasks (MacMillan et al., 2004; Evans, 2012), since they consider the position and context of the target landforms in the calculation. To be useful in landform extraction with MRS, LSPs should exhibit similar values within drumlins that are distinctly different from values outside. Based on these considerations the following five LSPs were chosen:

1. Convergence Index (*CI*): a measure of the extent to which steepest descent aspect (i.e., orientations of down-slope flow) for grid cells within a kernel converge on the center of the kernel (Köthe and Lehmeier, 1996); it is low when centered on topographic highs. *CI* is the only locally derived LSP chosen. Kernel sizes ranging from 3 × 3 to 31 × 31 cells were assessed visually, and 21 × 21 cells were found to produce reasonable results. At this scale the degree of convergence was relatively similar for the areas inside individual drumlin extents.
2. Slope Height (*SH*): provides the relative height above the closest modeled drainage accumulation (Böhner and Selige, 2006; Böhner and Antonic, 2009), and therefore, enhances drumlin relief.
3. Normalized Height (*NH*): defines the normalized difference between *SH* and the valley depth (i.e. the height below summit accumulation; Böhner and Selige, 2006; Böhner and Antonic, 2009). *NH* also enhances subtle drumlins.
4. SAGA Wetness Index (*SWI*): this is an indicator of water accumulation. It is computed based on slope values and the modified catchment area (Böhner and Selige, 2006). Higher values of *SWI* can be expected in areas of surface concavity, whereas surface convexities, such as drumlins, can be associated with lower values.
5. Vertical Distance to Channel Network (*VDTCN*): this describes the height above the channel network. It is measured as the relative height to an interpolated channel network base level (Bock and Köthe, 2008). This LSP is another example of a relative elevation layer that separates drumlin relief from the surrounding area. A similar layer has also been used in manual drumlin interpretation (Hillier and Smith, 2008).

The software used for deriving the five selected LSPs was SAGA (System for Automated Geoscientific Analyses; Olaya and Conrad, 2009; http://www.saga-gis.org).

**Fig. 2:**
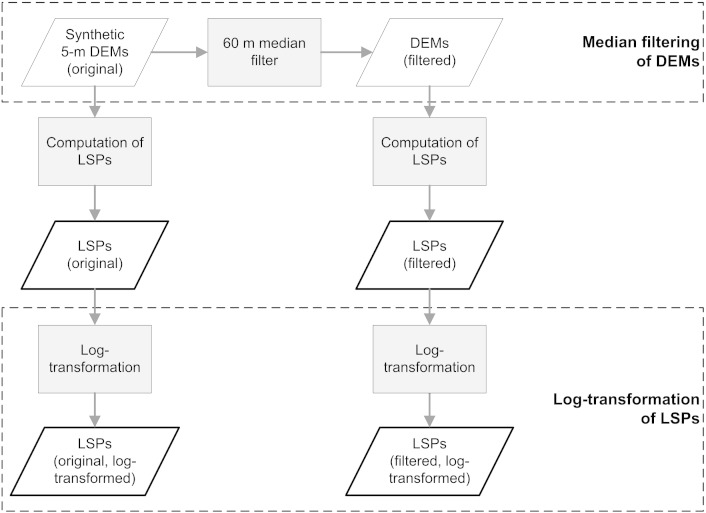
Workflow for computing four variants for each of the five selected land-surface parameters (LSPs) per synthetic DEM. The original synthetic 5-m DEM is pre-processed by a median filter. The two derived LSP variants are further log-transformed to produce another two variants.

The synthetic DEMs 1–5 included non-topographic features such as trees and building (see Fig. 1). To attempt to remove these features, a 60 m wide median filter was applied to the DEMs (Fig. 2). The filter width of 60 m was selected based on both visual (Hillier and Smith, 2012) and numerical (Hillier and Smith, 2014) analyses that suggest this value to be optimal for the area.

Since derived LSPs (except for *CI*) showed heavily skewed distributions, logarithmic transformation (hereafter “log-transformation”) was performed. Log-transformation had been recommended to use by Speight (1971), and is implemented in the geomorphometry toolbox for Esri ArcGIS (Reuter, 2009). As a result of this transformation, LSPs showed distributions close to normal (Speight, 1971).

Fig. 2 displays the workflow for deriving four variants of each LSP by applying a median filter to DEMs and log-transformation to LSPs. For each of the five DEMs a total of 20 LSP variants were computed.

### 3.3 Performance testing of multiresolution segmentation (MRS)

**Fig. 3:**
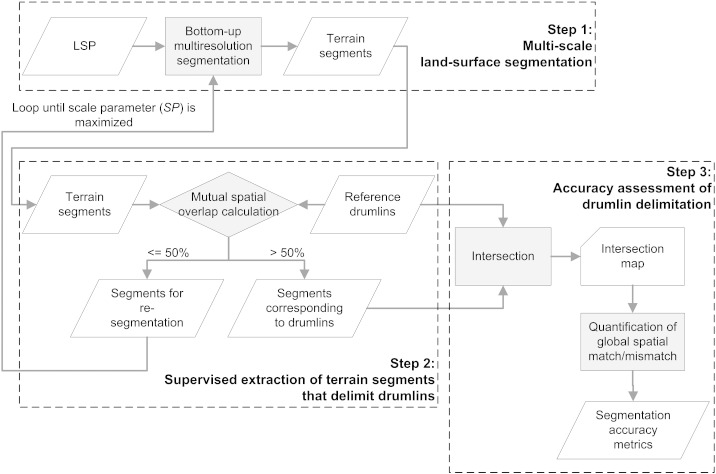
Workflow for performance testing of multiresolution segmentation (MRS) for drumlin delimitation based on land-surface parameters (LSPs).

Performance testing consisted of three steps (Fig. 3): (1) iterative multi-scale land-surface segmentation using MRS to partition the input LSP into consecutively larger terrain segments, (2) extraction of terrain segments that delimit synthetic drumlins by using a mutual spatial overlap threshold at 50%, and (3) drumlin segmentation accuracy assessment. The whole procedure was automated as a customized algorithm within an OBIA software, i.e. eCognition® (Trimble).

#### 3.3.1 Step 1: multi-scale land-surface segmentation

For drumlin delimitation by means of land-surface segmentation, the MRS algorithm is applied to derived LSPs and their variants. MRS employs iterative region-growing that is controlled by a user-specified relative homogeneity criterion known as “scale parameter” (*SP*). *SP* is a dimensionless parameter which specifies how homogeneous or heterogeneous segments are allowed to be within themselves on average. More specifically, the *SP* value defines the maximum allowed difference in “color homogeneity” and “shape homogeneity” between the initial segments and the segments resulting from the intended merge. “Color homogeneity” is measured based on grid cell values; “shape homogeneity” quantifies the deviation of the segment's 2D shape from a perfectly smooth or compact form. For the difference calculation the user has to set the relative importance of “color homogeneity” versus “shape homogeneity”, and further, for “shape homogeneity”, of “smoothness” versus “compactness” by specifying weights that in both cases sum up to 1. The higher the value of *SP*, the generally larger the resultant segments of the segmentation level. For technical details on the algorithm (including formulas) the reader is referred to Baatz and Schäpe (2000) and Benz et al. (2004).

Iterative MRS was applied to each LSP in a bottom-up manner by increasing the value of *SP* from 1 to 200 in an interval of 1. As a result, 200 segmentation levels consisting of increasingly larger terrain segments were generated for each LSP. A maximum *SP* of 200 was used, because up to this scale, the size of terrain segments was comparable to the size of synthetic drumlins. Weights for “shape homogeneity” and “color homogeneity” were set to 0.1 and 0.9, respectively, following previous object-based landform quantification studies (Drăguţ and Eisank, 2012; d'Oleire-Oltmanns et al., 2013). The selected weights imply that the creation of segments through MRS was mainly driven by the values of LSP grid cells, i.e. color, rather than by shape characteristics, i.e. smoothness and compactness.

#### 3.3.2 Step 2: extraction of terrain segments delimiting drumlins

This step was concerned with the identification of the set of terrain segments *S_i_* that delimit the set of reference drumlins *R_j_*, where *i* and *j* range from 1 to 173. eCognition® software allows automation of the extraction of *S_i_* through “image object links” (Trimble, 2013) which create virtual spatial connections between two independent set of objects (here: *S_i_* and *R_j_*) based on user-specified spatial overlap thresholds.

Synthetic drumlin polygons were imported into the software to be available for the spatial comparison with generated terrain segments. By using image object links, the following two spatial overlap criteria were evaluated for each terrain segment *S* of a segmentation level, and for each reference drumlin *R* (Weidner, 2008; Clinton et al., 2010):

∙ *A*_*R*_ ∩ *A*_*S*_ > *A*_*S*_/2: the overlap area has to be greater than half the area of the terrain segment, and
∙ *A*_*R*_ ∩ *A*_*S*_ > *A*_*R*_/2: the overlap area has to be greater than half the area of the reference drumlin.

If both criteria were fulfilled, meaning that *one* segment *S* existed that covered the majority of a reference drumlin *R* by more than half of its area, *S* was selected as a segment that corresponded to a drumlin, and added to the *S_i_* set with the current *SP* value stored as a property. Segments that did not meet the overlap criteria were re-segmented with the next higher *SP*. Then, the overlap criteria were re-evaluated for each newly derived terrain segment. Finally, the extracted *S_i_* set was intersected with the *R_j_* set to produce the “intersection map” (Fig. 3). This map depicts overlap and over-, and under-estimated areas of *S_i_* relative to *R_j_*.

**Fig. 4:**
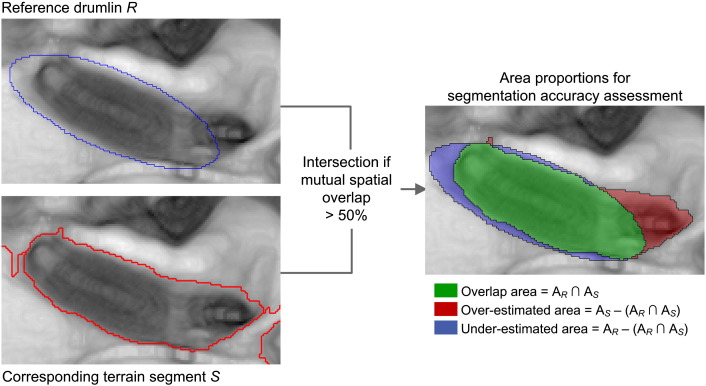
Illustration of image object linking for reference drumlin *R*_15_ (blue outline) in the synthetic DEM 3. Multiresolution segmentation (MRS) was based on the SAGA Wetness Index (*SWI*), as derived from the filtered synthetic DEM 3, and logarithmically transformed (see Section 3.2). At a scale parameter of 13, MRS delineated *S* (red outline). Since the mutual spatial overlap (green) between *R*_15_ and *S*, as evaluated through image object links, was larger than the threshold of 50%, *S* was selected as terrain segment delimiting *R*_15_, and denoted as *S*_15_. Over-estimated area is the area of *S*_15_ outside *R*_15_ (red); under-estimated area is the area of *R*_15_ not covered by S_15_ (blue).

Fig. 4 exemplifies the concept of image object links for one reference drumlin, *R*_15_. At an *SP* of 13, MRS yielded a terrain segment *S*_15_ that covered the majority of *R*_15_ by more than half of the segment's area. The measured spatial overlap of *S*_15_ with *R*_15_ at the scale of 13 was 83.5% (Table 1). At *SP* values lower than 13, *R*_15_ was intersected by multiple terrain segments that were all too small to fulfill the defined spatial overlap criteria of 50%.

#### 3.3.3 Step 3: accuracy assessment of drumlin delimitation

The area proportions between *S_i_* and *R_j_* were used to derive segmentation accuracy metrics for quantifying the quality of MRS-based drumlin delimitation. Various metrics for segmentation quality assessment have been proposed (Lucieer and Stein, 2002; Möller et al., 2007; Weidner, 2008; Liu et al., 2012). Clinton et al. (2010) provided a valuable review of the available measures.

The following three area-based measures were used (Table 2): “Quality Rate” (*QR*), “Area Fit Index” (*AFI*), and “Root Mean Square” (*D*), whereas *D* is a combined metric that is calculated as the sum of “Over Segmentation” (*OS*) and that of “Under Segmentation” (*US*) (Clinton et al., 2010). Except for *AFI*, the value range is from zero to 1; the closer the value is to zero, the better is the spatial match between reference objects and their largest overlapping segments, and the higher is the segmentation accuracy. Only *AFI* can reach negative values, mostly because of under-segmentation of the reference objects (Lucieer and Stein, 2002).

In addition, a fourth measure, the Miss Rate (*MR*), was introduced. *MR* relates the total number of reference objects $N_{R_{j}}$ to the number of undetected reference objects $N_{R_{m}}$ (i.e. objects without a corresponding segment) and is denoted as

(1) $$\mathrm{MR}=\frac{N_{R_{m}}}{N_{R_{j}}}.$$

The values of *MR* range from zero to 1. Values close to zero indicate a high rate of recovery of reference objects by the segmentation.

Moreover, the number of delimited drumlins, i.e. the number of terrain segments fulfilling the defined mutual spatial overlap threshold of 50% (*N*_S*i*_), was used as an evaluation criterion.

By applying the automated three-stepped workflow (Fig. 3) to the four variants of each of the five selected LSPs (see Fig. 2), 20 intersection maps were generated for each of the five synthetic DEMs, resulting in 100 maps in total. Obtained accuracies were compared to each other in order to evaluate the effects of median filtering of DEMs, and of log-transformation of LSPs on drumlin delimitation with MRS.

## 4 Results

### 4.1 Effects of median filtering of DEMs and log-transformation of LSPs on drumlin delimitation

**Fig. 5:**
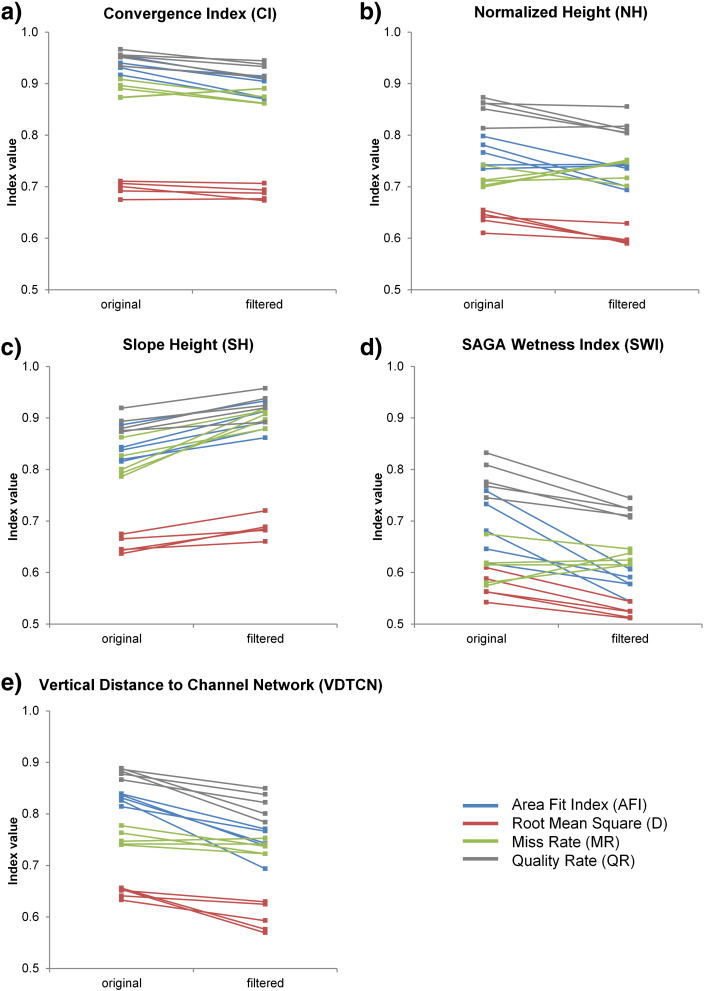
Effects of median filtering of DEMs on drumlin delimitation based on multiresolution segmentation. Segmentation accuracies for five LSPs (a to e) derived from the five original and filtered synthetic 5-m DEMs. Results for four metrics (color-coded).

Median filtering of DEMs was assessed by relating for each LSP the segmentation accuracies for the two variants derived from the five original and median filtered DEMs (Fig. 5). As can be seen in the figure, filtering of the DEM prior to the derivation of LSPs positively influenced segmentation accuracies for four LSPs (*CI*, *NH*, *SWI* and *VDTCN*). Improvements were the highest for *SWI* and *VDTCN* (up to 15%), and the lowest for *CI*. Only in case of *SH*, the variant based on the filtered DEMs yielded lower segmentation accuracies. Accuracy values for the same metric showed little variations across DEMs, regardless of LSP, suggesting that MRS is robust to differences in the locations and spatial distribution of synthetic drumlins.

**Fig. 6:**
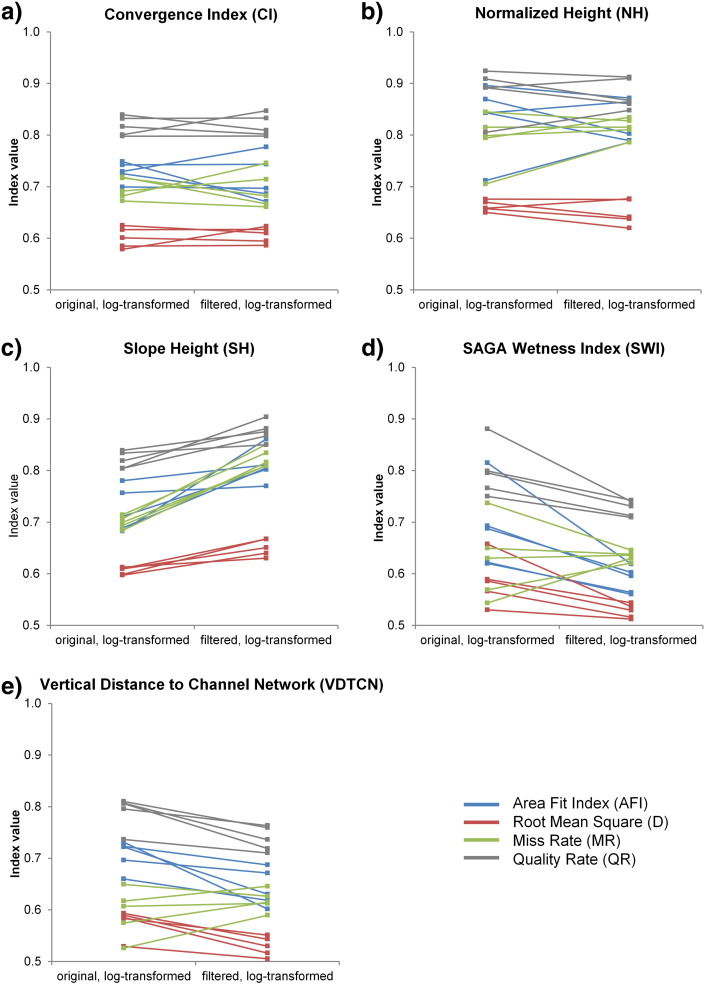
Drumlin delimitation accuracies based on multiresolution segmentation of log-transformed land-surface parameters (LSPs) derived from the five original and filtered synthetic 5-m DEMs. Results for five LSPs (a to e), and four metrics (color-coded).

In Fig. 6 results for the two log-transformed LSP variants are compared. In case of *SWI* and *VDTCN*, both of which are regionally derived LSPs, MRS performed slightly better on the log-transformed filtered variants than on the log-transformed original variants. Drumlin delimitation accuracies were the highest (i.e. indices values became the smallest) for log-transformed *SWI* and *VDTCN* (values between 0.75 and 0.5) derived from the filtered DEMs. No clear trend, i.e. increase or decrease, could be observed, when comparing the accuracies between the two log-transformed *CI* and *NH* variants. A comparison of log-transformed *SH* showed that lower accuracies were obtained for the variant that was based on the filtered DEMs.

**Fig. 7:**
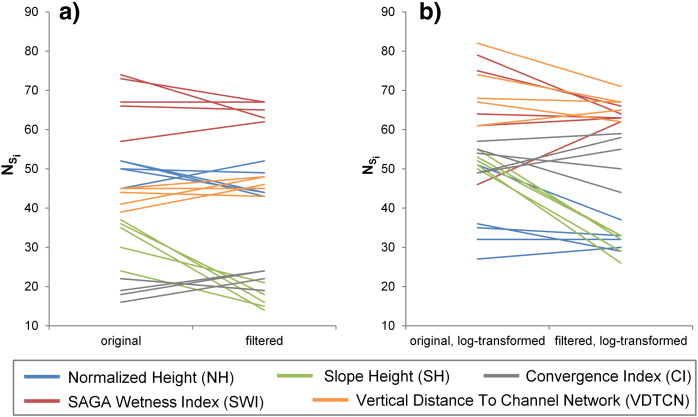
Effects of (a) median filtering of DEMs and (b) log-transformation of land-surface parameters (LSPs) on the number of terrain segments that spatially correspond to drumlins (*N*_S*i*_). The higher the number, the better drumlin recovery through multiresolution segmentation (MRS). Results for five LSPs (color-coded).

In general, MRS of *VDTCN* and *SWI* variants yielded the highest numbers of delimited drumlins (Fig. 7). Log-transformation of LSPs led to higher or at least similar numbers of delimited drumlins (Fig. 7b), as compared to their non-transformed variants (Fig. 7a), with improvements highest for *CI* (from values of ~ 15–25 to ~ 45–60) followed by *VDTCN*. The only exception from this rule was *NH*. The spread of *N_Si_* values over the five DEMs was higher for the two log-transformed variants. Median filtering of DEMs positively influenced drumlin delimitation for *CI* and *VDTCN*, whereas a negative impact was observed for the other three LSPs (Fig. 7a). As can be seen in Fig. 7b, for most LSPs there is no clear proof whether a log-transformation will improve drumlin delimitation or not. Application of log-transformation seems to depend on the underlying DEM. Only *SH* consistently showed better values for the variants derived from the original DEMs.

### 4.2 Intersection maps for SWI

**Fig. 8:**
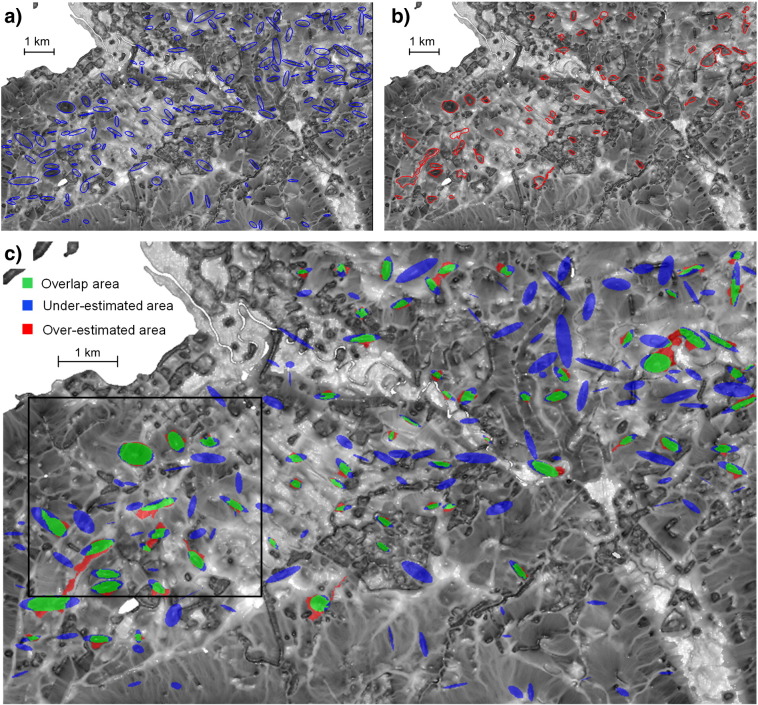
Maps showing results and accuracy of drumlin identification. (a) Reference drumlin polygons in the synthetic 5-m DEM 3 (in blue) and (b) their corresponding segments (in red) automatically identified by an iterative process of multiresolution segmentation of the log-transformed SAGA Wetness Index (*SWI*) as derived from the median filtered DEM 3. (c) Intersection map between (a) and (b) with classified areas of overlap, over- and under-estimation. Note that the map in (b) does not include segments outside of drumlins. Black rectangle in (c) shows the extent of the area for which results are displayed in Fig. 9.

**Fig. 9:**
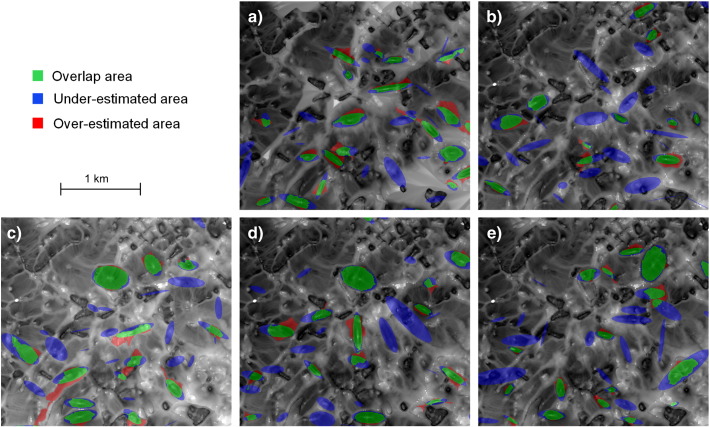
Detailed views of intersection results for the same sub-region of the study area (see black rectangle in Fig. 8c). Results based on multiresolution segmentation of the log-transformed SAGA Wetness Index (*SWI*) derived from the five median filtered synthetic 5-m DEMs (a to e).

Based on these systematic comparisons of segmentation accuracies and the numbers of delimited drumlins, MRS performs best with *VDTCN* and *SWI*. The overall best drumlin delimitation was achieved when segmenting log-transformed *SWI* based on the filtered DEM 3 (Fig. 8). From the 173 synthetic reference drumlins, 66 were successfully linked to their corresponding *SWI* segment when using a mutual spatial overlap criterion at 50%, whereas 107 drumlins were not detected in the course of iterative MRS and overlap evaluation. This caused the high proportion of under-estimated areas (blue in Fig. 8) in the intersection map. Nevertheless, results for the delimited drumlins appear promising, because the overlap area (green) shows that the spatial extents of delimited drumlins and reference drumlins matched quite well. The proportion of over-estimated areas (red) was relatively small, compared to the under-estimated parts. Similar conclusions can be drawn by analyzing the intersection maps for the log-transformed *SWI* variants derived from the remaining four median filtered synthetic DEMs shown in Fig. 9.

### 4.3 Scale parameter (SP)

The main parameter that controls MRS segmentation is *SP*. In order to know which *SP* values and ranges are optimal for drumlin delimitation with MRS, we analyzed the values that were stored as a property for each of the identified terrain segments.

Table 3 provides *SP* statistics for log-transformed variants of *SWI* and *VDTCN*, as derived from the median filtered DEMs. Commonly, the smallest synthetic drumlins were delimited at the lowest possible *SP* value of 1. The maximum *SP* value varied between 27 and 35 for *SWI*, and between 38 and 62 for *VDTCN*, depending on the underlying DEM. Median *SP* values for the same LSP were similar across DEMs.

**Fig. 10:**
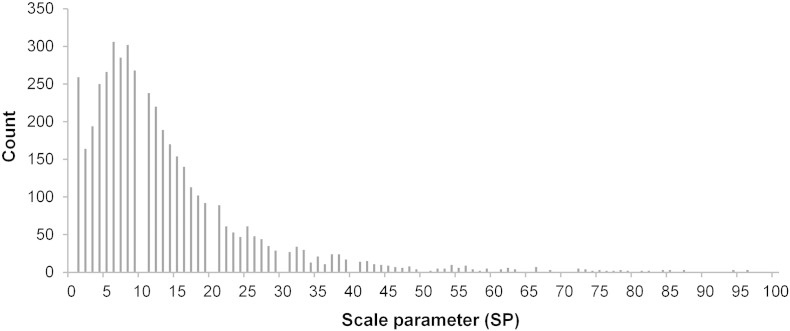
Frequency distribution of the scale parameter (*SP*) which yielded terrain segments that delimited synthetic drumlins. *SP* values obtained from 4609 terrain segments that delimited synthetic drumlins in 100 applications of the method. To ensure readability the 19 *SP* values above 100 (up to 189) were excluded from display.

The *SP* values and ranges for *SWI* and *VDTCN* were in line with the frequencies of drumlin delimitation relative to *SP* values (Fig. 10). The vast majority of the 4609 terrain segments that were associated with synthetic drumlins in 100 runs of the testing framework were delimited at *SP* values ranging from 1 to 50 (97%), with 80.5% delimited at scales from 1 to 20. This reflects the underlying distribution of drumlin sizes (Clark et al., 2009). The distribution is positively skewed and unimodal, reaching the main peak – corresponding to the maximum drumlin detection rate – between 5 and 10.

## 5 Discussion

This study presents a method for supervised testing of the performance of MRS, a widely-used region-growing segmentation algorithm, for the delimitation of synthetic drumlins from 5-m DEMs. The intention was to automatically find, for each synthetic reference drumlin, the *one* spatially best matching terrain segment. This was ensured by constraining the mutual spatial overlap between synthetic drumlins and terrain segments to be larger than 50%. Only one segment could potentially meet these overlap conditions. Therefore, the proposed method identified one-to-one relations between drumlins and segments. Other types of relations (one-to-many, many-to-one, many-to-many) were disregarded. Analysis of one-to-many relations, where each drumlin is represented by several terrain segments (over-segmentation), will only be superior to the proposed approach, if optimal rules for the merging of drumlin-intersecting terrain segments can be found.

The supervised testing framework allowed for the analysis of the effects of median filtering of DEMs and log-transformation of LSPs on the performance of drumlin delimitation. The four segmentation accuracy metrics proved to be valuable tools for quantifying the spatial agreement and discrepancy between the results of multi-scale segmentation and the reference drumlin maps. The testing framework is automated, and can potentially be applied to any exercise where segments are to be detected based upon available reference objects.

The highest drumlin delimitation accuracies were reached with log-transformed LSPs that were based on median filtered DEMs. Then, up to 50% of drumlins were satisfactorily delimited by single terrain segments. Regionally derived LSPs such as *VDTCN* and *SWI* produced more accurate drumlin delimitation than locally derived LSPs such as *CI*. This is because regional LSPs are computed based on topographically meaningful regions and linear structures rather than on morphologically irrelevant fixed-sized kernel (Olaya, 2009). For instance, *VDTCN* is computed by evaluating the catchment area and the height above the channel network (Bock and Köthe, 2008). In contrast to fixed-sized regular kernels, the evaluated regions per grid cell are different in size, depending on the structure of the land surface.

Ideally, MRS for drumlin delimitation should be multi-scalar with different *SP* values. The performance tests on *VDTCN* and *SWI* showed that no optimal *SPs* for MRS-based drumlin delimitation exist. The *SP* value where most drumlins are delimited by MRS depends on the LSP and its characteristics such as value range and local variability.

It is worth noting that MRS-based drumlin recovery of about 50% is within the same range of accuracy as that achieved by manual interpretation; for this work an average recovery rate of 43% was achieved by one expert and one non-expert using the same synthetic DEMs. The analysis of DEMs representing the current land surface, as used in this study, is not always appropriate for drumlin quantification, since the ancient surface may have been smoothed due to postglacial modification, including sedimentation. Finlayson (2013) suggested that removing the sedimentation layer from the DEMs may improve computer-based drumlin delimitation.

Pre-processing through filtering helped in reducing the clutter that was present in the original synthetic DEMs. Filtering was a compromise between reduction of DEM noise and preservation of drumlin reliefs (Hillier and Smith, 2012; Hillier and Smith, 2014). It has been reported that British drumlins are shallower than drumlins in other regions of the world (Spagnolo et al., 2012). This also holds true for the selected study area: about 50% of the synthetic drumlins exhibited a maximum relief below 5 m. Since the filtered DEMs still included some noise, it can be concluded that the 60 m median filter was adequate for smoothing the DEMs, a finding noted by Hillier and Smith (2014). However, the remaining noise will have disturbed the smooth character of some drumlins relief. By definition, MRS is sensitive to such surface modifications (Baatz and Schäpe, 2000). The resultant terrain segments in these areas are – to a certain degree – arbitrarily defined, and deviate from the form and size of the synthetic drumlins. As a result, these segments rarely meet the defined mutual spatial overlap criteria, and hence, these drumlins are not delimited by MRS. However, the delimitation performance of MRS on LSPs derived from filtered DEMs is generally better than on LSPs derived from unfiltered DEMs.

**Fig. 11:**
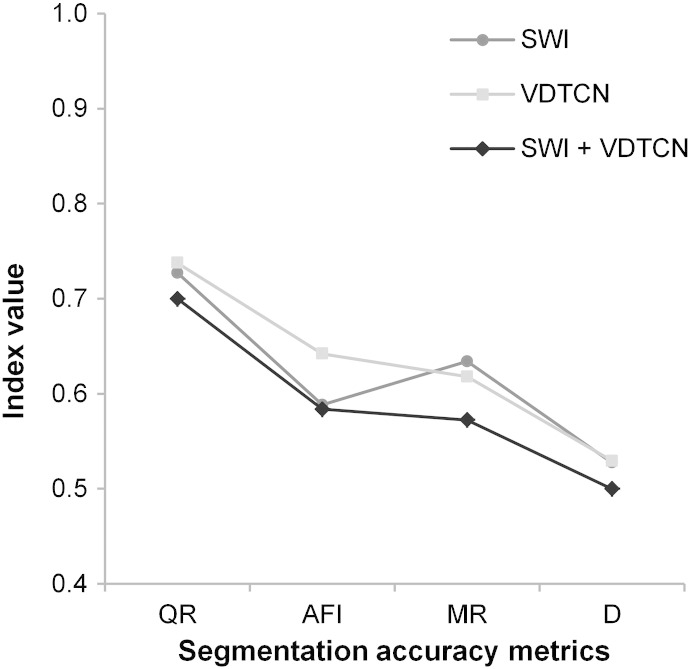
Accuracy of drumlin delimitation by means of the multiresolution segmentation of log-transformed SAGA Wetness Index (*SWI*) and/or Vertical Distance To Channel Network (*VDTCN*), as derived from the five median filtered synthetic 5-m DEMs. Graphs present average values of four metrics: Quality Rate (*QR*), Area Fit Index (*AFI*), Miss Rate (*MR*), and Root Mean Square (*D*).

There are several limitations in the current study: firstly, for all tests a fixed mutual overlap threshold of 50% (Weidner, 2008; Clinton et al., 2010) was used. Secondly, only one segmentation algorithm, i.e. MRS, was analyzed among others. Thirdly, the experiments were conducted for individual LSPs, but not for multiple LSPs in combination. Using only one LSP might be insufficient in delineating landforms such as drumlins (Dikau, 1989). However, when combining LSPs for MRS, they can be weighted according to their drumlin detection performance in our tests. A first test on the combined use of LSPs for MRS-based drumlin delimitation shows that accuracy values for the four metrics slightly improve, as compared to the values obtained from MRS of single LSPs (Fig. 11).

**Fig. 12:**
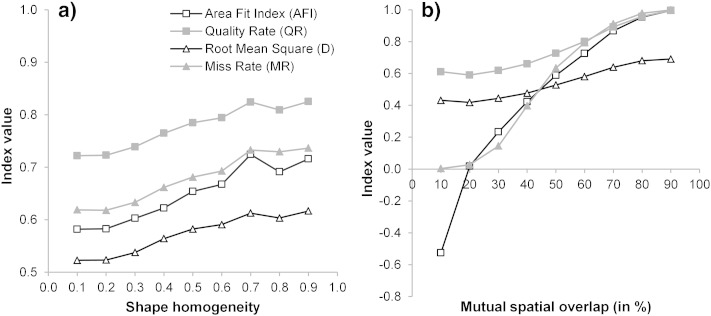
Effects of changing (a) the shape homogeneity and (b) the mutual spatial overlap threshold on four drumlin segmentation accuracy metrics. Results obtained from multiresolution segmentation of the log-transformed SAGA Wetness Index (*SWI*) derived from five median filtered synthetic 5-m DEMs.

A preliminary assessment of the impact of changed mutual spatial overlap, as well as of altered “shape homogeneity” on MRS performance indicates that the selected values of 50% and 0.1, respectively, are suitable for drumlin delimitation (Fig. 12). Tests were based on the best performing LSP variants, i.e. the log-transformed *SWI* values as derived from the median filtered DEMs. When changing the mutual spatial overlap threshold to values lower than 50%, the overall quality of drumlin delimitation improves (Fig. 12b). This is because drumlins that have been missed previously are detected at lower thresholds, but overlapping areas significantly reduce with lower thresholds. Setting the overlap threshold to values higher than 50% reduces segmentation accuracies, since fewer drumlins are detected, and those detected are over-estimated by terrain segments. Hence, the threshold of 50% is a good compromise between over- and under-estimation of drumlins. Also, the threshold determining the relative importance of shape over color in MRS (i.e. the “shape homogeneity”) has been adequately defined (Fig. 12a): highest drumlin segmentation accuracies were obtained at the selected value of 0.1. Higher weights of “shape homogeneity” resulted in less accurate results. Further tests are needed to confirm these preliminary findings.

Usually, OBIA involves two steps: an initial segmentation and a subsequent classification (Blaschke, 2010). This study has only dealt with segmentation, and quantification of segmentation error relevant to the accuracy of the classification (Clinton et al., 2010). Once MRS delineates terrain segments that are similar to the size and shape of target landforms, it is possible to correlate segment properties with the characteristics of landforms. High correlations indicate properties that may be well-suited to object-based landform classification. The ultimate goal of such correlation analysis is to support the design of semantic landform models that hold structural information about landform-defining properties (Eisank et al., 2011).

Although we have the data and tools at hand, automating the process of manual landform interpretation remains challenging due to questions of scale, ambiguous landform definitions, and vagueness in the spatial extent of various landforms (Mark and Smith, 2004; Evans, 2012). In contrast to previous landform quantification studies (e.g. Anders et al., 2011), we used synthetic landforms that represent objective and reliable reference data. In this way, one major source of uncertainty (i.e. the subjectivity of manually delineated references) was removed. In combination with the use of multiple synthetic DEMs that included the same population of drumlins at different locations, we created an ideal experiment for testing drumlin delimitation algorithms such as MRS more widely and effectively. We believe that the conclusions drawn in this study are transferable to other areas, landform categories, and DEMs at comparable spatial resolutions.

## 6 Conclusions

This paper assessed the potential of a widely used image segmentation algorithm, multiresolution segmentation (MRS), in delimiting drumlins based on land-surface parameters (LSPs) derived from synthetic DEMs. The devised testing scheme is based on an automated evaluation of mutual spatial overlaps between MRS-derived terrain segments and idealized drumlin polygons. Terrain segments are identified as drumlin-delimiting segments when they cover more than half of a drumlin area by more than half of their own area. The following conclusions can be drawn from the results obtained from 100 tests:

∙ Drumlin delimitation by means of MRS tends to work better on log-transformed LSPs that were regionally derived from median filtered DEMs.
∙ Both lower and upper values of scale parameter were suggested for MRS-based drumlin delimitation. Effective value ranges depend on LSP.
∙ The rates of drumlin recovery can be compared to those achieved by manual interpretations. Delimited drumlins correspond reasonably to the size and shape of idealized drumlins.
∙ MRS appears robust to changes in the location and spatial distribution of idealized drumlins in the DEM.

The outcomes of this study are important for optimizing drumlin delimitation by means of MRS, especially for areas, where no reference data are at hand. Synthetic DEMs, as used in this study, represent idealized landforms, and are objective and reliable datasets for supervised testing of landform quantification methods. The above conclusions will therefore be valid in other areas, and might be applicable to different landform categories and DEMs.

## Figures and Tables

**Table 1:** Spatial overlap statistics for the synthetic drumlin *R*_15_, as used in Fig. 4. Values were obtained during iterative bottom-up multiresolution segmentation. The area of *R*_15_ is 3653 grid cells (5 m spatial resolution).

Scale parameter (*SP*)	No. of overlapping terrain segments	Area of the largest overlapping terrain segment (in grid cells)	Overlap of the largest overlapping segment with *R*_15_ (in grid cells)	% Overlap relative to *R*_15_
1	62	119	119	3.3
2	37	248	248	6.8
3	27	314	314	8.6
4	17	1021	1021	28.1
5	11	1021	1021	28.1
6	10	1021	1021	28.1
7	9	1021	1021	28.1
8	7	1021	1021	28.1
9	4	1021	1021	28.1
10	4	1021	1021	28.1
11	4	1021	1021	28.1
12	4	1021	1021	28.1
13	1	3650	3033	83.5

**Table 2:** Overview of the selected segmentation accuracy metrics. $A_{R_{j}}$ indicate the total area of reference drumlins *R_j_*; $A_{S_{i}}$ define the total area of corresponding terrain segments *S_i_*.

Segmentation accuracy metric	Formula	Original reference	Applied in
Quality rate	$\mathrm{QR}=1-\frac{A_{R_{j}}\cap A_{S_{i}}\;}{A_{R_{j}}\cup A_{S_{i}}}$	Winter (2000)	Weidner (2008), Liu et al. (2012) and Whiteside et al. (2012)
Area fit index	$\mathrm{AFI}=\frac{A_{R_{j}}-A_{S_{i}}\;}{A_{R_{j}}}$	Lucieer and Stein (2002)	Neubert et al. (2008), Clinton et al. (2010) and Bar Massada et al. (2012)
Over segmentation	$\mathrm{OS}=1-\frac{A_{R_{j}}\cap A_{S_{i}}}{A_{R_{j}}}$	Clinton et al. (2010)	Johnson et al. (2012) and Liu et al. (2012)
Under segmentation	$\mathrm{US}=1-\frac{A_{R_{j}}\cap A_{S_{i}}}{A_{S_{i}}}$	Clinton et al. (2010)	Johnson et al. (2012) and Liu et al. (2012)
Root mean square	$D=\sqrt{\frac{{\left(\mathrm{OS}\right)}^{2}+{\left(\mathrm{US}\right)}^{2}}{2}}$	Clinton et al. (2010)	Johnson et al. (2012) and Liu et al. (2012)

**Table 3:** Scale parameter (*SP*) statistics for the SAGA Wetness Index (*SWI*) and Vertical Distance To Channel Network (*VDTCN*); both land-surface parameters (LSPs) were derived from five median filtered synthetic 5-m DEMs and log-transformed.

	*SWI*					*VDTCN*				
	DEM 1	DEM 2	DEM 3	DEM 4	DEM 5	DEM 1	DEM 2	DEM 3	DEM 4	DEM 5
*SP*_min_	1	1	2	1	1	1	1	1	1	1
*SP*_max_	27	35	31	27	28	54	41	38	62	54
*SP*_median_	7	6	7	7	7	12	13	13	14	13
